# Long-Term Epidemiology of *Streptococcus pneumoniae* Serogroup 6 in a Region of Southern Europe with Special Reference to Serotype 6E

**DOI:** 10.1371/journal.pone.0149047

**Published:** 2016-02-10

**Authors:** José M. Marimón, María Ercibengoa, Esther Tamayo, Marta Alonso, Emilio Pérez-Trallero

**Affiliations:** 1 Microbiology Department, Hospital Universitario Donostia-Instituto Biodonostia, San Sebastián, Spain; 2 Biomedical Research Center Network for Respiratory Diseases (CIBERES), San Sebastián, Spain; 3 Faculty of Medicine, University of the Basque Country, UPV/EHU, San Sebastián, Spain; Faculdade de Medicina de Lisboa, PORTUGAL

## Abstract

*Streptococcus pneumoniae* serotype 6E has recently been described, but its long-term epidemiology is not well known. From 1981–2013, 704 serogroup 6 clinical isolates were obtained in Gipuzkoa, Basque Country, Spain. All invasive and one in four non-invasive isolates were included. Overall, 75, 97, 51 and 45 serotypes 6A, 6B, 6C and 6E isolates, respectively, were detected. No serotype 6D isolates were identified. The prevalence of serotypes 6E and 6B, but not that of serotypes 6A and 6C, declined after the introduction of pneumococcal conjugate vaccines. Serotype 6E isolates showed the highest resistance rate. Most serotype 6E isolates were ST90.

## Introduction

Among the nearly 100 different pneumococcal serotypes currently described, *Streptococcus pneumoniae* serogroup 6 serotypes have been extensively studied, especially serotype 6B [1]. When pneumococcal capsular genes were described in 2006 [2], new serotypes (6C, 6D [3, 4] and 6E [5]) were added to the classical serotypes (6A and 6B) in serogroup 6. Because serotype 6E has been described so recently, there is scarce information on its clinical burden and epidemiological trends over long periods.

The main objective of this work was to describe the clinical and microbiological characteristics of serotype 6E pneumococci in our region during a 33-year period.

## Materials and Methods

This retrospective study was conducted in San Sebastian, Basque Country, northern Spain. Serotyping was performed with the Quellung reaction using polyclonal antisera (Statens Serum Institut, Copenhagen, Denmark) and multiplex PCR [6].

Serotype 6E was investigated in all serogroup 6 isolates from invasive infections and in a random selection of one in four non-invasive serogroup 6 isolates to accomplish a similar number of invasive and non-invasive isolates. Serotype 6E isolates were detected by PCR amplification and sequencing of the four open reading frames (*orf1* to *orf4*) present between the *dexB* and *wzy* genes [5]. Genotyping was performed on all serotype 6E isolates by PFGE and MLST [7, 8]. Antibiotic susceptibility was determined by the broth microdilution method according to the Clinical and Laboratory Standards Institute (CLSI) [9].

The epidemiological and clinical data of patients with serotype 6E infection, including the 30 days mortality in invasive infections, were recorded. For statistical analysis, categorical variables were compared using the chi-square test or the Fisher exact test when appropriate.

This study was approved by the Ethical Committee for Clinical Research of the Health Area of Gipuzkoa with a waiver of informed consent documentation since this was a retrospective study and patients' identities were safeguarded.

## Results

Between 1981 and 2013, 2,082 invasive and 7,595 non-invasive *S*. *pneumoniae* isolates were serotyped. The present investigation included all 124 invasive and 144 (one in four of the 577) non-invasive serogroup 6 isolates. The serotypes found among these 268 isolates were as follows: 75 (28.0%) 6A; 97 (36.2%) 6B, 51(19.0%) 6C and 45 (16.8%) 6E (Table 1). There were no serotype 6D isolates. Serotype 6E, consisting of 10 invasive and 35 non-invasive isolates, caused mainly respiratory infections (10 cases of pneumonia, 10 COPD exacerbations, and 6 lower respiratory infections). Of the patients with serotype 6E invasive infections, three had cancer (stomach, leukemia and lymphoma); two patients had chronic bronchitis (one of them also with non-alcoholic cirrhosis and diabetes); two were HIV coinfected patients, one had senile dementia and mild respiratory insufficiency and myocardial sclerosis; in the other two patients no underlying diseases were recorded. The first serotype 6E isolate was collected in 1981 from the pleural fluid of a 45-year-old man with pneumonia.

**Table 1:** Serotype distribution by disease and diagnostic sample among 268 serogroup 6 isolates in Gipuzkoa, northern Spain, 1981–2012.

Disease	Sample	Serotype				Total
		6A	6B	6C	6E	
Meningitis (n = 22)						
	CSF	8	8	3	-	19
	Blood	-	2	1	-	3
Sepsis (primary bacteremia) (n = 43)						
	Blood	10	19	8	6	43
Peritonitis and arthritis (n = 9)						
	Blood	1	2	-	-	3
	Sterile fluid	-	4	2	-	6
Pneumonia (n = 80)						
	Blood	13	19	10	3	45
	Bronchial secretion	9	11	4	6	30
	Pleural fluid	1	2	1	1	5
Other LRI (COPD exacerbation, …) (n = 59)	Bronchial secretion	15	16	12	16	59
Otitis media and conjunctivitis (n = 50)	Middle ear fluid and conjunctival exudate	18	13	8	11	50
Wound infections, dermal abscess, … (n = 5)	Other samples	-	1	2	2	5
Total isolates		75	97	51	45	268

CSF: cerebrospinal fluid; LRI: lower respiratory infections; COPD: chronic obstructive pulmonary disease

Serotype 6E caused fewer invasive infections than serotypes 6A, 6B or 6C (p<0.01) but had a similar prevalence to the other three serotypes in non-invasive infections (Table 1).

When we compared two consecutive 12-year periods, before (1990–2001) and after (2002–2013) the introduction of the heptavalent pneumococcal conjugate vaccine (PCV7) in our region, we found that the prevalence of invasive infections caused by serotypes 6E (9/57 versus 0/60) and 6B (34/57 versus 18/60) but not that of serotype 6A (11/57 versus 20/60) declined (p ≤0.001). Serotype 6C invasive infections increased (3/57 versus 22/60, p <0.001). As a cause of global, invasive and non-invasive infections, isolates of serotypes 6B (p<0.015) and 6E (p<0.001) decreased in the post-PCV7 period, and serotypes 6A (p = 0.03) and 6C (p< 0.001) increased.

Two patients with serotype 6E invasive infection died (20%): a 95-year-old man with primary bacteraemia and an 86-year man with COPD and pneumococcal pneumonia. Mortality due to serotypes 6A, 6B and 6C causing invasive disease was 4/33 (12.1%), 4/59 (6.8%) and 4/25 (16.0%), with three deaths (two serotype 6C and one 6A) being due to meningitis.

The mean and median age of patients infected with serotype 6E was 46 years and 57 years, respectively (range 25 days to 95 years) and 34% were women. The mean/median age of patients infected with serotypes 6A, 6B and 6C were 39.4/50 years, 44.5/56 years, and 44.7/55 years, respectively.

All 45 serotype 6E isolates had initially been serotyped as 6B with the Quellung reaction. Overall, 15 different STs (ST90 = 20) were identified, of which five were first labelled in this study (ST10528, 10529, 10530, 10531, and 10535). eBURST analysis revealed three different clonal complexes (CC156, CC315, CC2779). The most prevalent was CC156, comprising 36 of the 45 serotype 6E isolates that were also grouped together by PFGE. ST90 was the most frequent genotype both in invasive and non-invasive isolates, ST96, ST273, ST2779, ST3999, ST5127, ST5531, ST10528, ST10529, and ST10530 being found only among non-invasive isolates and ST315 only in invasive isolates.

Among serogroup 6 isolates, serotype 6E isolates showed the higher resistance rates. The highest percentage of resistance, with significant differences, was obtained between serotype 6E and serotypes 6A and 6C against beta-lactams: penicillin, amoxicillin and cefotaxime; macrolides and lincosamides; sulfamethoxazole trimethoprim (SXT); tetracycline; and chloramphenicol (Table 2). No differences in resistance were found between serotype 6E and 6B isolates except for SXT and chloramphenicol, against which 6E isolates also showed the highest resistance rate (p< 0.003). Resistance to fluoroquinolones and rifampin was infrequent and was similar among isolates of the four serogroup 6 serotypes. Overall, 86.7% (39/45) serotype 6E isolates were multiresistant (non-susceptible to three or more antimicrobial classes).

**Table 2:** Number and percentage on non-susceptible isolates against thirteen antimicrobials among 268 serogroup 6 isolates.

	Susceptible breakpoint	Serotypes							
	(μg/mL)	6A		6B		6C		6E	
		(n = 75)		(n = 97)		(n = 51)		(n = 45)	
		N°	(%)	N°	(%)	N°	(%)	N°	(%)
Oral benzylpenicillin	≤ 0.06	13	(17.3)	63	(66.0)	12	(23.5)	36	(80.0)
Parenteral benzylpenicillin	≤ 2	0	(0.0)	8	(8.2)	0	(0.0)	2	(4.4)
Amoxicillin	≤ 2	0	(0.0)	14	(14.4)	0	(0.0)	7	(15.6)
Cefotaxime	≤ 1	0	(0.0)	5	(5.2)	0	(0.0)	4	(8.9)
Erythromycin	≤ 0.25	19	(25.3)	73	(75.3)	16	(31.4)	27	(60.0)
Clindamycin	≤ 0.25	14	(18.7)	70	(72.2)	14	(27.5)	25	(55.6)
Tetracycline	≤ 1	23	(30.7)	67	(69.1)	9	(17.6)	33	(73.3)
Trimethoprim-sulfamethoxazole	≤ 0.5/9.5	22	(29.3)	44	(45.4)	4	(7.8)	33	(73.3)
Chloramphenicol	≤ 4	10/73*	(13.7)	11/95*	(11.6)	0	(0.0)	15	(33.3)
Rifampin	≤ 1	12/73*	(16.4)	2	(2.1)	4	(7.8)	2	(4.4)
Ciprofloxacin	≤ 2	1	(1.3)	1	(1.0)	2	(3.9)	2	(4.4)
Levofloxacin	≤ 2	0	(0.0)	0	(0.0)	1	(2.0)	2	(4.4)
Vancomycin	≤ 1	0	(0.0)	0	(0.0)	0	(0.0)	0	(0.0)

* Susceptibility to all antibiotics was not available in two 6A and 6B isolates

## Discussion

*S*. *pneumoniae* serotype 6E has a worldwide distribution [5, 10]. However, the current prevalence rates of serotype 6E among serogroup 6 isolates vary widely between different countries, with the highest rates being found in Asian regions [10, 11]. Due to the high rates of antibiotic resistance among serotype 6E isolates, if prevalence studies of serotype 6E are done in selected multiresistant isolates, a higher rate of 6E isolates will be expected. The present series has the advantage of being obtained from unselected invasive and non-invasive serogroup 6 isolates.

Although most initial studies considered that the introduction of PCV7 had no influence on the prevalence of serotype 6E [10, 12], a recent work demonstrated that serotype 6B antibodies induced by PCV7 had a protective effect against serotype 6E pneumococci [13]. Our population-based data study also supports this latter hypothesis, as serotype 6E was isolated more frequently before the introduction of PCV7 in late 2001.

Genotyping showed a clonal distribution of serotype 6E isolates in our region, with CC156 (ST90) being the most common. This clonal distribution is very similar to that of other countries and regions [10, 12–14] with the exception of China, where ST982 was the most common ST, followed by ST90 [14].

## Conclusions

In Gipuzkoa, northern Spain, serotype 6E pneumococci were isolated in all age groups causing a wide variety of diseases, except meningitis. Most serotype 6E isolates were multiresistant and the decrease in all serotype 6E infections after the introduction of PCV7 in our country supports the hypothesis that the serotype 6B included in PCV formulations confers cross-protection against this long-standing but recently described serotype.
